# Risk factors for liver dysfunction and their clinical importance after gastric cancer surgery

**DOI:** 10.1038/s41598-024-58644-0

**Published:** 2024-04-06

**Authors:** Shutaro Sumiyoshi, Takeshi Kubota, Takuma Ohashi, Keiji Nishibeppu, Jun Kiuchi, Hiroki Shimizu, Tomohiro Arita, Yusuke Yamamoto, Hirotaka Konishi, Ryo Morimura, Yoshiaki Kuriu, Atsushi Shiozaki, Hisashi Ikoma, Hitoshi Fujiwara, Eigo Otsuji

**Affiliations:** Division of Digestive Surgery, Department of Surgery, Kyoto Prefectural University of Medicine, 465 Kajii-cho, Kamigyo-ku, Kyoto, 602-8566 Japan

**Keywords:** Gastroenterology, Medical research, Oncology, Risk factors

## Abstract

Postoperative hepatobiliary enzyme abnormalities often present as postoperative liver dysfunction in patients with gastric cancer (GC). This study aimed to identify the risk factors for postoperative liver dysfunction and their clinical impact after GC surgery. We retrospectively analyzed the data of 124 patients with GC who underwent laparoscopic or robotic surgery at Kyoto Prefectural University of Medicine between 2017 and 2019. Twenty (16.1%) patients with GC developed postoperative liver dysfunction (Common Terminology Criteria for Adverse Events (CTCAE) version 5.0 ≥ Grade 3). Univariate analyses identified robotic surgery as a risk factor for postoperative liver dysfunction (*P* = 0.005). There was no correlation between the postoperative liver dysfunction status and postoperative complications or postoperative hospital stays. Patients with postoperative liver dysfunction did not have significantly worse overall survival (*P* = 0.296) or recurrence-free survival (*P* = 0.565) than those without postoperative liver dysfunction. Robotic surgery is a risk factor for postoperative liver dysfunction; however, postoperative liver dysfunction does not affect short or long-term outcomes.

## Introduction

Gastric cancer (GC), the fifth most common cancer worldwide, has the third highest mortality rate^1^. Surgical treatment, with or without chemotherapy, remains the primary option for patients with stage-based localized GC. Currently, minimally invasive surgical approaches for GC, such as laparoscopic gastrectomy (LG) and robotic gastrectomy (RG), are widely applied^2^. According to a meta-analysis of randomized controlled trials, LG yields better surgical safety, lower operative morbidity, less trauma, and faster recovery than open gastrectomy (OG)^3^. However, phase III clinical trials have shown that LG is more frequently associated with elevated serum aspartate aminotransferase (AST) and alanine aminotransferase (ALT) levels than OG^4^.

The association between postoperative complications and poor long-term outcomes has been reported in various surgical fields^5–8^. In patients with GC, several studies have confirmed the relationship between postoperative complications after gastrectomy and poor oncological prognosis^9–11^. However, postoperative liver dysfunction is not included in the Clavien-Dindo classification used to assess postoperative complications^12^. Therefore, the prognostic impact of postoperative liver dysfunction, which is increasing with the spread of minimally invasive surgical approaches in GC surgery, needs further elucidation. This study retrospectively identified the putative risk factors for postoperative liver dysfunction and investigated the prognostic impact of postoperative liver dysfunction in patients with GC who underwent radical resection.

## Materials and methods

### Patients

**Figure 1:**
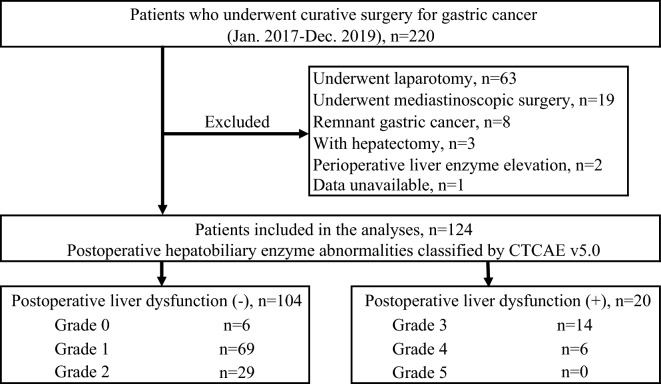
Flowchart for selecting and classifying patients. Of 220 consecutive patients with GC who underwent curative surgery, 96 were excluded, and 124 were eligible for this study. Twenty patients were classified into the postoperative liver dysfunction-positive group, whereas the remaining 104 patients were classified into the postoperative liver dysfunction-negative group. *GC* Gastric cancer.

This study was conducted following the ethical principles of Kyoto Prefectural University of Medicine and the Declaration of Helsinki. Informed consent was obtained from all participants through opt-outs on our hospital website. The Ethical Review Board of the Kyoto Prefectural University of Medicine approved the experimental protocol (ERB-C-1414-1). Data from 220 consecutive patients who underwent curative resection for GC at Kyoto Prefectural University of Medicine between 2017 and 2019 were retrospectively analyzed. The patient underwent a gastrectomy and lymph node dissection following the guidelines of the Japanese Society of Gastric Cancer^13^. Patients were excluded if they had preoperative hepatobiliary enzyme abnormalities (Common Terminology Criteria for Adverse Events (CTCAE) version 5.0 > Grade 1), underwent laparotomy or mediastinoscopic surgery, had remnant GC, underwent gastrectomy with hepatectomy, or were unavailable. Finally, 124 patients with GC were included in the study (Fig. 1).

Data on patient characteristics, pathological and surgical findings, and postoperative clinical courses were obtained from the institution's medical records and databases. Physical examinations and blood tests, including those for tumor markers, were performed every 3 months, and computed tomography was performed every 6 months. Further treatment in cases of recurrence was decided based on the patient’s consent, condition, and available evidence at that time.

Notably, various clinical factors have been examined to determine their associations with postoperative liver dysfunction in patients with GC. Serum levels of AST, ALT, alkaline phosphatase (ALP), and total bilirubin (T-BIL) were evaluated in all patients. The upper limits of normal (ULN) at our institution were AST 30 IU/L, ALT 42 IU/L, ALP 322 IU/L, and T-BIL 1.5 mg/dL. Hepatobiliary enzyme abnormalities were assessed using the CTCAE’s diagnostic criteria recommended by the Council for International Organizations of Medical Sciences (Supplementary Table S1). Serum hepatobiliary enzyme levels were routinely measured preoperatively, on postoperative days 1, 3, and 7, and days after based on the postoperative course. Postoperative liver dysfunction was characterized based on hepatobiliary enzyme abnormalities of ≥ Grade 3. Specifically, AST, ALT, and ALP values greater than 5 times ULN (AST ≥ 150 IU/L, ALT ≥ 210 IU/L, ALP ≥ 1610 IU/L) and T-BIL greater than 3 times ULN (T-BIL ≥ 4.5 mg/dL) are considered Grade 3.

### Liver retraction methods

In all cases, the left liver lobe was retracted using a Nathanson liver retractor (Cook Medical, Indiana, USA) subsequent to completing dissection of the greater curvature and subpyloric lymph nodes. The Nathanson retractor was positioned proximate to the porta hepatis, under the lateral segment of the liver, following insertion near the xiphoid process. A silicone disc (Hakko Corporation, Nagano, Japan) was used to prevent hepatic decompression due to the Nathanson liver retractor, and the position of the Nathanson liver retractor was altered hourly.

### Statistical analysis

Data were analyzed using JMP version 10 (ASA Institute, Cary, NC, USA). Chi-square and Fisher’s exact probability tests were used to compare categorical variables between groups, whereas Student’s t-tests and Mann–Whitney U tests were used for unpaired continuous data. Survival curves were estimated using the Kaplan–Meier method, and differences were evaluated using the log-rank test. Statistical significance was set at *P* < 0.05.

## Results

### Changes in postoperative liver enzyme levels

Of the 124 patients who underwent gastrectomy, 20 (16.1%) developed postoperative liver dysfunction (Table 1). Perioperative changes in AST and ALT levels in patients with and without postoperative liver dysfunction are presented in Table 2. Serum AST and ALT levels were significantly higher in the postoperative liver dysfunction-positive group than in the postoperative liver dysfunction-negative group on postoperative days 1 and 3 (*P* = 0.001). AST levels peaked on postoperative day 1, and ALT levels peaked on postoperative day 3 and then gradually decreased.

**Table 1:** The clinicopathological characteristics of patients with GC.

Variables	Overall	(n = 124)
Sex		
Female	53	(43%)
Male	71	(57%)
Age		
Mean ± SD (years)	67.4 ± 10.8	
Preoperative BMI		
Mean ± SD (kg/m^2^)	22.1 ± 3.3	
Depth of tumor		
T1	102	(82%)
T2	12	(10%)
T3	7	(6%)
T4	3	(2%)
Lymph node metastasis		
N0	102	(82%)
N1	15	(12%)
N2	5	(4%)
N3	2	(2%)
Stage		
I	107	(86%)
II	13	(11%)
III	4	(3%)
Surgical approach		
Laparoscopic	99	(80%)
Robotic	25	(20%)
Surgical procedure		
Distal gastrectomy	91	(73%)
Proximal gastrectomy	25	(20%)
Total gastrectomy	8	(7%)
Operative time		
Median (min)	310 (169–657)	
Estimated blood loss		
Median (g)	24 (0–610)	
Postoperative complications^a^		
Grade 0	98	(79%)
Grade I	4	(3%)
Grade II	17	(14%)
Grade III	4	(3%)
Grade IV	1	(1%)
Postoperative liver enzyme abnormalities^b^		
Grade 0, 1, 2	104	(84%)
Grade 3, 4, 5	20	(16%)

*BMI* Body mass index, *GC* Gastric cancer, *SD* Standard deviation.

^a^Clavien–Dindo classification.

^b^Common Terminology Criteria for Adverse Events.

**Table 2:** Changes in hepatobiliary enzymes in patients with or without postoperative liver dysfunction.

Variables	Postoperative liver dysfunction				Univariate^a^
	( +)	(n = 20)	( −)	(n = 104)	*P* value
AST, (median [range])					
Preoperative	21.0	(14–56)	22.0	(14–69)	0.506
POD1	373.5	(150–2521)	48.0	(18–129)	**0.001**
POD3	119.0	(26–765)	28.0	(10–118)	**0.001**
POD7	30.5	(14–170)	34.0	(12–95)	0.94
ALT, (median [range])					
Preoperative	17.5	(7–84)	18.0	(6–95)	0.825
POD1	315.0	(119–1269)	40.0	(12–159)	**0.001**
POD3	321.0	(56–1041)	31.0	(6–209)	**0.001**
POD7	100.0	(14–285)	41.0	(8–137)	**0.001**
ALP, (median [range])					
Preoperative	206.5	(150–409)	213.0	(79–763)	0.417
POD1	173.0	(112–310)	156.0	(86–336)	0.405
POD3	163.5	(101–289)	154.0	(81–679)	0.532
POD7	225.5	(129–495)	212.5	(100–797)	0.417
T-Bil, (median [range])					
Preoperative	0.65	(0.43–1.25)	0.70	(0.33–1.95)	0.959
POD1	0.96	(0.64–2.20)	0.99	(0.39–2.86)	0.742
POD3	0.92	(0.67–3.15)	0.88	(0.27–3.09)	0.081
POD7	0.67	(0.32–4.46)	0.60	(0.23–2.08)	0.501

Significant values are in [bold].

*AST* aspartate aminotransferase, *ALP* alkaline phosphatase, *ALT a*lanine aminotransferase, *POD* postoperative day, *T-Bil* total bilirubin.

^a^Univariate analysis included Chi squared and Fisher’s exact probability tests.

### Clinicopathological factors of postoperative liver dysfunction

Twenty patients had postoperative liver dysfunction (Grade 3, n = 14; Grade 4, n = 6). Univariate analysis revealed that postoperative liver dysfunction was significantly associated with RG (*P* = 0.005). There were no correlations between the postoperative liver dysfunction status and sex, age, preoperative body mass index, liver disease, any other comorbidities, tumor, node, metastasis status, surgical procedure, lymphadenectomy, reconstruction, operative time, blood loss, intraoperative blood transfusion, presence of replaced left hepatic artery, dissection of replaced left hepatic artery, postoperative complications, or postoperative hospital stay (Table 3).

**Table 3:** Univariate analyses of the potential risk factors for postoperative liver dysfunction.

Factor	Variables	Postoperative liver dysfunction				Univariate^a^
		( +)	(n = 20)	( −)	(n = 104)	*P* value
Sex	Female/male	10/10		43/61		0.622
Age, years (mean ± SD)		65.2 ± 10.8		67.8 ± 10.8		0.245
Preoperative BMI, kg/m^2^ (mean ± SD)		22.6 ± 4.0		22.1 ± 3.1		0.828
Any comorbidity	Yes/no	14/6		67/37		0.799
Liver disease	Yes/no	2/18		6/98		0.481
Pathological T	< T3/ ≥ T3	4/16		6/98		0.083
Pathological N	N0/ ≥ N1	1/19		21/83		0.123
Pathological TNM stage	I/ ≥ II	16/4		91/13		0.890
Surgical procedure	DG/TG or PG	14/6		77/27		0.826
Surgical approach	Laparoscopy/Robot	11/9		88/16		**0.005**
With cholecystectomy	Yes/no	2/18		5/99		0.614
Lymphadenectomy	D1 or D1 + /D2	18/2		97/7		0.637
Reconstruction	B-I/B-II/R-Y/other	10/0/5/5		44/0/40/20		0.721
Operative time, min (median [range])		328.0 (180–535)		307.0 (169–657)		0.520
Estimated blood loss, g (median [range])		2.5 (0–225)		27.5 (0–610)		0.329
Intraoperative blood transfusion	Yes/no	0/20		0/104		
Replaced left hepatic artery	Present/absent	0/20		10/94		0.363
Dissection of replaced left hepatic artery	Yes/no	0/20		6/98		0.588
Postoperative complications	CD grade ≥ 2	2/18		20/84		0.523
	CD grade ≥ 3a	0/20		5/99		0.317
Postoperative hospital stay, days (median [range])		10.0 (8–18)		11.0 (7–30)		0.400

Significant values are in [bold].

*BMI* Body mass index, *B-I* Billroth-I, *B-II* Billroth-II, *CD* Clavien–Dindo, *DG* Distal gastrectomy, *PG* Proximal gastrectomy, *R-Y* Roux-en-Y, *SD* Standard deviation, *TG* Total gastrectomy.

^a^Univariate analysis included Chi squared and Fisher’s exact probability tests.

### Differences in clinicopathological factors in LG and RG

Ninety-nine patients (79.8%) were classified into the laparoscopic surgery group, and the remaining 25 (20.2%) into the robotic surgery group. Univariate analysis revealed that robotic surgery was significantly associated with a longer operative time (*P* < 0.001) and less frequent complications of Clavien–Dindo classification ≥ Grade 2 (*P* = 0.022). There were no correlations between the surgical approach and sex, age, body composition, histological type, tumor size, tumor depth, staging, operative time, blood loss, intraoperative blood transfusion, presence of replaced left hepatic artery, dissection of replaced left hepatic artery, or postoperative complications (Table 4).

**Table 4:** Main characteristics of the patients with laparoscopic and robotic gastrectomy.

Factor	Variables	Surgical approach		Univariate^a^
				*P* value
		Laparoscopy (n = 99)	Robot (n = 25)	L versus R
Sex	Female/male	40/59	13/12	0.297
Age, years (mean ± SD)		69.0 ± 11.5	65.0 ± 7.0	0.123
Preoperative BMI, kg/m^2^ (mean ± SD)		22.4 ± 3.4	22.5 ± 3.8	0.782
Any comorbidity	Yes/no	61/38	20/5	0.103
Liver disease	Yes/no	6/93	2/23	0.662
Pathological T	< T3/ ≥ T3	8/91	2/23	0.628
Pathological N	N0/ ≥ N1	20/79	2/23	0.241
Pathological TNM stage	I/ ≥ II	85/14	22/3	0.781
Surgical procedure	DG/TG or PG	71/28	20/5	0.688
With cholecystectomy	Yes/no	7/92	0/25	0.357
Lymphadenectomy	D1 or D1 + /D2	92/8	24/1	0.685
Reconstruction	B-I/B-II/R-Y/other	43/0/35/21	11/0/10/4	0.801
Operative time, min (median [range])		300.0 (169–657)	365.0 (254–535)	** < 0.001**
Estimated blood loss, g (median [range])		25.0 (0–610)	7.0 (0–210)	0.195
Intraoperative blood transfusion	Yes/no	0/99	0/25	
Replaced left hepatic artery	Present/absent	8/91	2/23	1.000
Dissection of replaced left hepatic artery	Yes/no	6/93	0/25	0.347
Postoperative liver dysfunction	Yes/no	36/63	13/12	0.174
Postoperative complications	CD grade ≥ 2	21/78	1/24	**0.022**
	CD grade ≥ 3a	5/94	0/25	0.129
Postoperative hospital stay, days (median [range])		11.0 (7–30)	10.0 (8–14)	0.342

Significant values are in [bold].

*BMI* Body mass index, *B-I* Billroth-I, *B-II* Billroth-II, *CD* Clavien–Dindo, *DG* Distal gastrectomy, *PG* Proximal gastrectomy, *R-Y* Roux-en-Y, *SD* Standard deviation, *TG* Total gastrectomy.

^a^Univariate analysis included Chi squared and Fisher’s exact probability tests.

### Analysis of prognostic factors

**Figure 2:**
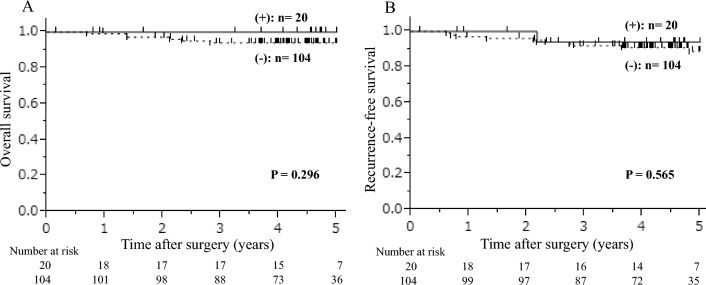
Overall (**A**) and recurrence-free (**B**) survival curves of patients with GC with or without postoperative liver dysfunction. The patients were classified into two groups based on the presence or absence of postoperative liver dysfunction. Groups with postoperative liver dysfunction are indicated with solid lines, and groups without postoperative liver dysfunction are indicated with dashed lines. Patients with postoperative liver dysfunction did not have a significantly poorer prognosis in the 5-year OS and RFS than those without postoperative liver dysfunction. *GC* Gastric cancer, *OS* Overall survival, *RFS* Recurrence-free survival.

The median follow-up period was 4.61 years (interquartile range 3.78–5.11). Figure 2 shows the overall survival (OS) and recurrence-free survival (RFS) curves for patients with GC with and without postoperative liver dysfunction. The 5-year OS and RFS rates in the 20 patients with postoperative liver dysfunction were 100.0% and 95.0%, respectively. In contrast, the 5-year OS and RFS rates of the 104 patients without postoperative liver dysfunction were 94.2% and 90.4%, respectively. Patients with postoperative liver dysfunction did not have a significantly worse OS (*P* = 0.296) or RFS (*P* = 0.565) than those without postoperative liver dysfunction.

## Discussion

The following novel findings were observed in this study. First, robotic surgery is a risk factor for postoperative liver dysfunction after gastrectomy for patients with GC. Second, postoperative liver dysfunction did not affect short or long-term outcomes in patients with GC.

Postoperative liver dysfunction after laparoscopic surgery has also been reported in laparoscopic cholecystectomy^14^ and laparoscopic colorectal resection^15^, suggesting that insufflation reduces blood flow to the liver and may cause elevated liver enzyme levels and functional impairment. Conversely, risk factors for liver dysfunction in GC surgery include the dissection of the replaced left hepatic artery and the retraction of the lateral segment of the liver associated with the dissection^16,17^. This study identified robotic surgery as a risk factor for liver dysfunction after GC surgery. To our knowledge, this is the first study to include a cohort treated with RG and to examine the estimated risk factors for postoperative liver dysfunction. RG significantly decreased the postoperative complication rate while prolonging the operation time compared with LG. However, these clinicopathological and surgical factors were not risk factors for postoperative liver dysfunction, and dissection of the replaced left hepatic artery was not significantly different in this study. This suggests that other factors may also contribute to liver dysfunction in patients undergoing RG. At our institution, the pneumoperitoneum pressure and liver retraction methods used in RG were similar to those used in LG; however, the head-up angle was steeper in RG. In the case of the method using a retractor fixed to the surgical bed (or operating table), an excessive head-up position may cause the patient's body to slide down (Supplementary Fig. S1), increasing the pressure on the liver (Supplementary Fig. S2).

Surgical robots have articulated forceps, tremor-filtering capabilities, and high-resolution three-dimensional images. Therefore, RG is expected to overcome LG’s limitations and enable more meticulous surgeries. Notably, many retrospective studies have revealed that RG is associated with fewer postoperative complications, less intraoperative blood loss, and longer operative times, consistent with the results of the present study^2^. When RG was introduced at our institution for the first time in 2015, we used the 15° head-up position and a Nathanson liver retractor^18^ for liver retraction. However, after experiencing severe liver dysfunction (C-D grade 4a) in 2016, the intraoperative position was changed to a 13° head-up and a closed leg position with a plantar plate to prevent patient displacement. In addition, we used a Nathanson retractor with a silicone disc^19^ and changed the position of the liver retraction every hour. Although our patients have not experienced severe liver dysfunction since then, liver enzyme elevations are still common in RG procedures. Our study cohort included only patients who underwent RG after implementation of these measures. There have been no reports of postoperative liver dysfunction in robotic surgery; however, prolonged liver retraction time due to the prolonged surgical time, liver retraction methods, and positioning differences, such as the head-up angle, could have an effect. Moreover, surgeons may adjust liver positioning, the risk of ischemic liver dysfunction remains, and prolonged operation times could exacerbate this risk.

An association between postoperative liver dysfunction and long-term prognosis has been reported in colorectal cancer^20^; however, its association with GC is unclear. Notably, previous reports have described an association between preoperative liver enzyme abnormalities and poor prognosis, suggesting that chronic liver inflammation affects the GC prognosis^21,22^. Our study found no association between postoperative liver dysfunction and long-term prognosis. This may be because most cases of postoperative liver dysfunction in GC surgeries are due to focal hepatic injury caused by mechanical liver retraction and are unassociated with preexisting chronic inflammation of the liver. In this study, postoperative liver dysfunction did not affect short-term outcomes such as postoperative hospital stay. However, there have been reports of liver necrosis caused by retraction-related compression intraoperatively^23^. Therefore, liver retraction for adequate dissection is acceptable in GC surgery; however, measures to prevent liver injury are critical.

This study has some limitations, including its retrospective nature and single-center sample size. Liver dysfunction is a concern that should not be tolerated unconditionally; therefore, large cohort studies are needed to validate these findings before their clinical application. In addition, several perioperative factors may influence postoperative liver dysfunction. However, completely removing the influence of perioperative factors on postoperative liver status was difficult.

In conclusion, our study found that robotic surgery is a risk factor for postoperative liver dysfunction; however, postoperative liver dysfunction itself does not affect short or long-term outcomes.

### Supplementary Information

Supplementary Information.

## Data Availability

All data generated or analyzed during this study are included in this published article and its Supplementary Information files.
