# Supplementary material for: Risk factors for liver dysfunction and their clinical importance after gastric cancer surgery
Source: Sci Rep. 2024 Apr 6;14:8076. doi: 10.1038/s41598-024-58644-0 (PMC10997756; doi:10.1038/s41598-024-58644-0)
Supplement: Supplementary file 1 — Supplementary Information. [file 41598_2024_58644_MOESM1_ESM.pdf]

Supplementary Table S1. Definition of hepatobiliary enzyme abnormalities in Common Terminology Criteria for Adverse Events (CTCAE) version 5.0

MedDRA Code	MedDRA SOC	CTCAE Term	Grade 1	Grade 2
10001551	Investigations	Alanine aminotransferase increased	>ULN - 3.0 x ULN if baseline was normal; 1.5 - 3.0 x baseline if baseline was abnormal	>3.0 - 5.0 x ULN if baseline was normal; >3.0 - 5.0 x baseline if baseline was abnormal
10001675	Investigations	Alkaline phosphatase increased	>ULN - 2.5 x ULN if baseline was normal; 2.0 - 2.5 x baseline if baseline was abnormal	>2.5 - 5.0 x ULN if baseline was normal; >2.5 - 5.0 x baseline if baseline was abnormal
10003481	Investigations	Aspartate aminotransferase increased	>ULN - 3.0 x ULN if baseline was normal; 1.5 - 3.0 x baseline if baseline was abnormal	>3.0 - 5.0 x ULN if baseline was normal; >3.0 - 5.0 x baseline if baseline was abnormal
10005364	Investigations	Blood bilirubin increased	>ULN - 1.5 x ULN if baseline was normal; >1.0 - 1.5 x baseline if baseline was abnormal	>1.5 - 3.0 x ULN if baseline was normal; >1.5 - 3.0 x baseline if baseline was abnormal

MedDRA Code	MedDRA SOC	CTCAE Term	Grade 3	Grade 4
10001551	Investigations	Alanine aminotransferase increased	>5.0 - 20.0 x ULN if baseline was normal; >5.0 - 20.0 x baseline if baseline was abnormal	>20.0 x ULN if baseline was normal; >20.0 x baseline if baseline was abnormal
10001675	Investigations	Alkaline phosphatase increased	>5.0 - 20.0 x ULN if baseline was normal; >5.0 - 20.0 x baseline if baseline was abnormal	>20.0 x ULN if baseline was normal; >20.0 x baseline if baseline was abnormal
10003481	Investigations	Aspartate aminotransferase increased	>5.0 - 20.0 x ULN if baseline was normal; >5.0 - 20.0 x baseline if baseline was abnormal	>20.0 x ULN if baseline was normal; >20.0 x baseline if baseline was abnormal
10005364	Investigations	Blood bilirubin increased	>3.0 - 10.0 x ULN if baseline was normal; >3.0 - 10.0 x baseline if baseline was abnormal	>10.0 x ULN if baseline was normal; >10.0 x baseline if baseline was abnormal

MedDRA Code	MedDRA SOC	CTCAE Term	Definition
10001551	Investigations	Alanine aminotransferase increased	A finding based on laboratory test results that indicate an increase in the level of alanine aminotransferase (ALT or SGPT) in the blood specimen.
10001675	Investigations	Alkaline phosphatase increased	A finding based on laboratory test results that indicate an increase in the level of alkaline phosphatase in a blood specimen.
10003481	Investigations	Aspartate aminotransferase increased	A finding based on laboratory test results that indicate an increase in the level of aspartate aminotransferase (AST or SGOT) in a blood specimen.
10005364	Investigations	Blood bilirubin increased	A finding based on laboratory test results that indicate an abnormally high level of bilirubin in the blood. Excess bilirubin is associated with jaundice.

Supplementary Figure S1.
Change in patient's body position pre and post-operatively at 15º head-up position

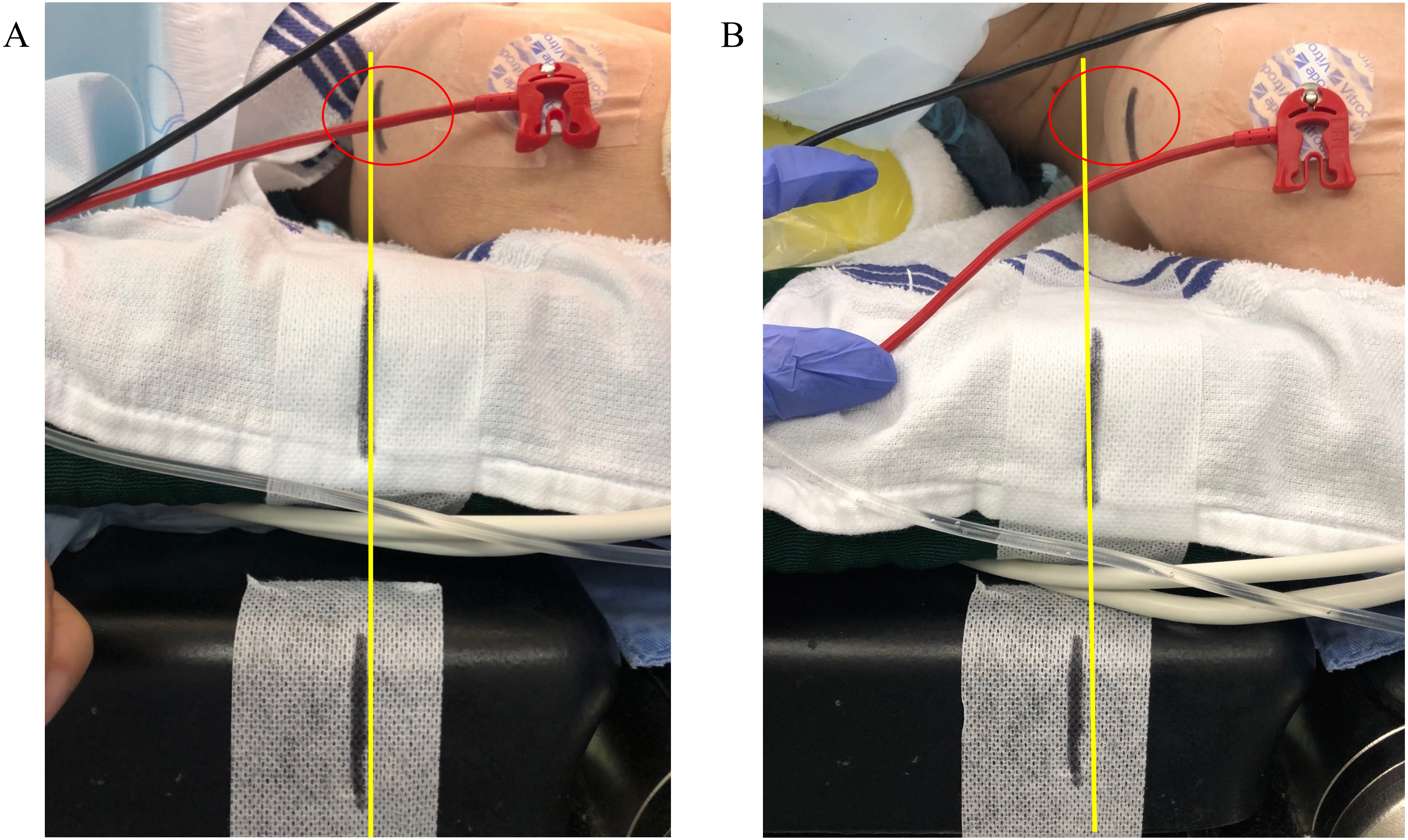

Notably, some patients moved caudally from the bed position markings preoperatively (A) to postoperatively (B).

Supplementary Figure S2. Liver dysfunction due to excessive retraction of the liver

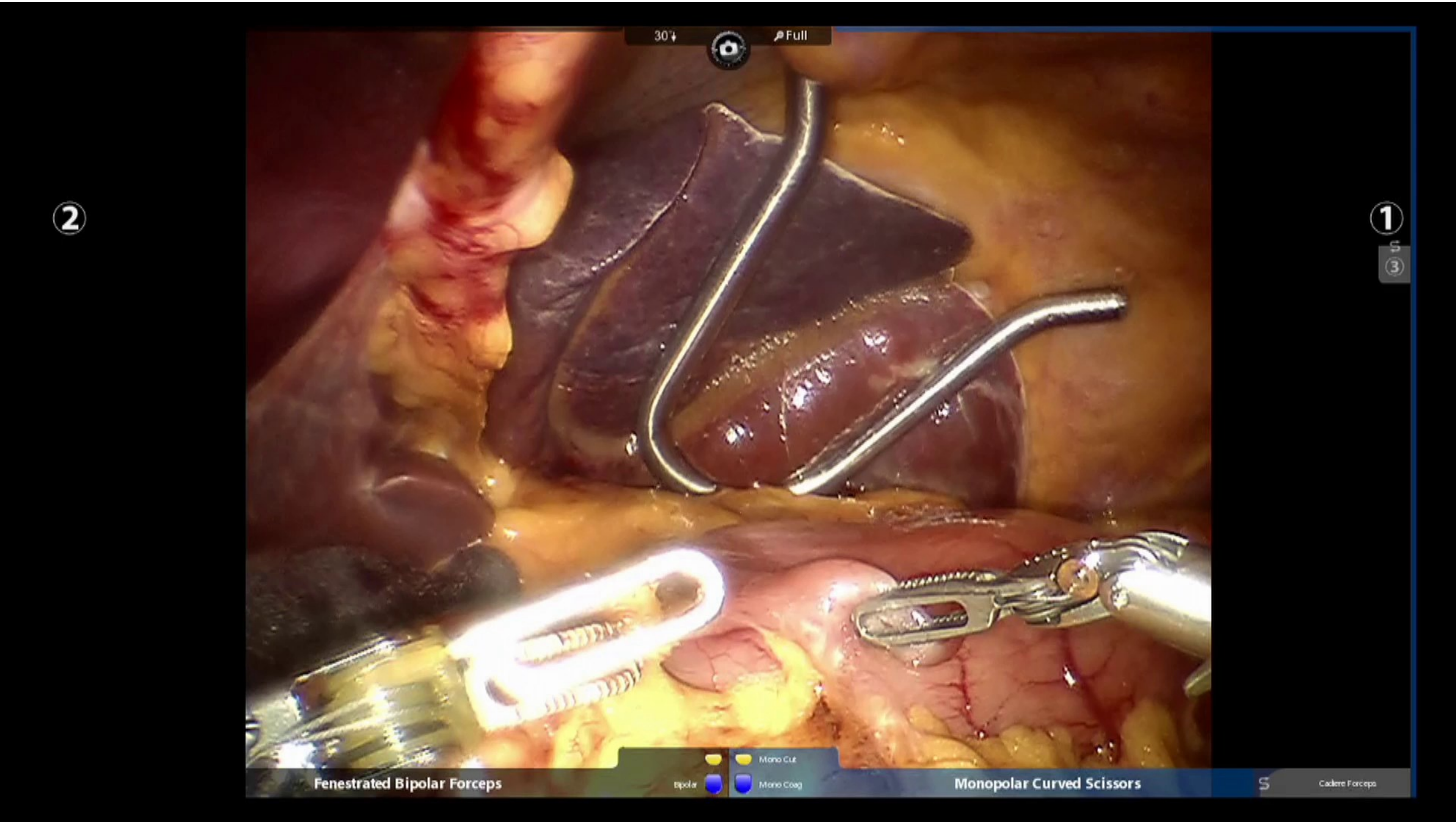

Liver retraction using a Nathanson retractor resulted in color changes on the liver surface during RG. In the present case, postoperative liver dysfunction caused DIC and required ICU management.

RG, robotic gastrectomy; DIC, disseminated intravascular coagulation; ICU, intensive care unit
